# Activating PER Repressor through a DBT-Directed Phosphorylation Switch 

**DOI:** 10.1371/journal.pbio.0060183

**Published:** 2008-07-29

**Authors:** Saul Kivimäe, Lino Saez, Michael W Young

**Affiliations:** Laboratory of Genetics, The Rockefeller University, New York, New York, United States of America; University of Geneva, Switzerland

## Abstract

Protein phosphorylation plays an essential role in the generation of circadian rhythms, regulating the stability, activity, and subcellular localization of certain proteins that constitute the biological clock. This study examines the role of the protein kinase Doubletime (DBT), a *Drosophila* ortholog of human casein kinase I (CKI)ɛ/δ. An enzymatically active DBT protein is shown to directly phosphorylate the *Drosophila* clock protein Period (PER). DBT-dependent phosphorylation sites are identified within PER, and their functional significance is assessed in a cultured cell system and in vivo. The *per* ^S^ mutation, which is associated with short-period (19-h) circadian rhythms, alters a key phosphorylation target within PER. Inspection of this and neighboring sequence variants indicates that several DBT-directed phosphorylations regulate PER activity in an integrated fashion: Alternative phosphorylations of two adjoining sequence motifs appear to be associated with switch-like changes in PER stability and repressor function.

## Author Summary

Most proteins involved in circadian transcriptional feedback loops undergo reversible chemical modifications (called phosphorylation) that regulate their activity in a time-of-day–dependent manner. Doubletime (DBT), a *Drosophila* kinase, phosphorylates the circadian transcriptional repressor PERIOD (PER). Mutations of *dbt* shorten or lengthen the period of circadian behavioral rhythms, or abolish the rhythms altogether in flies. A mutation of the human ortholog of *dbt*, *casein kinase I* (*CKI*)δ, has been associated with certain forms of a heritable sleep disorder. The disorder may reflect altered activity of a human PER protein, as the syndrome can also be caused by mutation of a CKIɛ/δ phosphorylation site within PER2. In this study, we locate DBT-directed phosphorylation sites in the *Drosophila* PER protein, including a DBT target region of PER that was previously shown to regulate DBT activity. Two PER domains within this region appear to serve as alternative targets for DBT. Phosphorylation of the upstream domain seems to suppress phosphorylation elsewhere in the region, producing a stable PER protein with little activity as a transcriptional repressor. However, when phosphorylation of the upstream domain is blocked, downstream DBT targets appear to be phosphorylated, producing a highly active, but short-lived repressor. Our results suggest that ordered patterns of DBT-directed phosphorylation contribute to the timing of PER's function and disappearance, and thus influence the pace of the circadian clock.

## Abstract 3

Two phosphorylation domains in*Drosophila* PERIOD protein interact in a switch-like fashion with each other and the kinase DOUBLETIME to regulate PER's stability and activity as a transcriptional repressor in the circadian transcriptional feedback loop.

## Introduction

Circadian rhythms (∼24-h rhythms) in physiology and behavior are observed in almost all phyla. In fungi, plants, and animals, cell autonomous, autoregulatory gene and protein interactions promote circadian molecular oscillations. Most of the genes and proteins that make up such clocks are not conserved between phyla, suggesting that circadian clocks may have evolved independently in bacteria, fungi, plants. and animals. Notable exceptions are the protein kinases CK1 and CK2. The former is known to play a central role in both animal and fungal clocks, while CK2 has been shown to be essential for animal and plant circadian rhythmicity [1]. Although in certain cases cycling clock gene transcription can be eliminated without suppressing circadian rhythmicity, protein phosphorylation appears to be essential for persistent clock function [2].

The circadian clock of Drosophila melanogaster is composed in part of two interconnected feedback loops [3]. In the *period/timeless* (*per/tim*) loop, CLOCK (CLK) and CYCLE (CYC), two transcription factors of the bHLH/PAS superfamily, heterodimerize and activate *per* and *tim* transcription during the afternoon and early evening. In turn, after nuclear translocation and binding to CLK/CYC heterodimers, PER, and possibly TIM, repress their own transcription during late night and early morning [4]. In the *Clock* (*Clk*) loop, circadian transcription of *vrille* (*vri*) and of *Par domain protein 1*ɛ (*Pdp1*ɛ) is driven by CLK/CYC heterodimers, and subsequently, VRI negatively regulates the transcription of *Clk*. Still later, Pdp1 renews expression of *Clk* [5]. *Pdp1* may also have a role in mediating circadian clock output [6].

Most proteins involved in these transcriptional feedback loops are phosphorylated in a time-of-day–dependent manner, and three kinases, casein kinase I (CKI)ɛ/δ/Doubletime (DBT), CK2, and GSK-3/Shaggy (SGG), have been identified as essential clock components in *Drosophila*. Importantly, a mutation of the human ortholog of DBT, CKIδ, has been associated with certain forms of a heritable disorder of sleep, familial advanced sleep phase syndrome (FASPS; [7]). The disorder may reflect altered phosphorylation of PER2, as FASPS has also been associated with mutation of a putative CKIɛ/δ phosphorylation site within PER2 [8]. Preliminary studies of the prevalence of FASPS indicate that the disorder may affect as much as 0.3% of the United States population (C. R. Jones and L. J. Ptacek, unpublished data).

DBT is constitutively expressed at the mRNA and protein level, and is an essential gene for fly development, regulating cell survival and proliferation [9–11]. Loss of DBT activity is associated with hypophosphorylation and stabilization of PER, indicating its role in determining the rate of PER degradation [12]. DBT has been shown to physically interact with PER in vitro and in vivo, and to create a stable complex with PER throughout the circadian cycle [9,10]. PER that has been phosphorylated by DBT is recognized by the Slimb protein [13]. Slimb is a component of the Skp1/Cullin/F-box protein (SCF) ubiquitin ligase complex, which marks proteins for proteosomal degradation in a phosphorylation-dependent manner [14]. Enhanced PER degradation in the cytoplasm is predicted to delay nuclear translocation of both PER and TIM, and to thus affect the period of circadian rhythms [12].

The mutation *dbt* ^S^, associated with an amino acid substitution (proline to serine at residue 47 [P47S]), shortens period length by about 6 h. *dbt* ^L^, with an amino acid substitution of isoleucine for methionine at residue 80 (M80I), lengthens period to 29 h. A third mutation, *dbt* ^AR^, is associated with a change from histidine 126 to tyrosine (H126Y) and causes arrhythmia. PER protein in this mutant is hypophosphorylated [15]. Each of these mutations maps to the kinase domain of DBT [16]. The short- and long-period alleles of DBT enhance or attenuate, respectively, PER degradation in the nucleus, further demonstrating the importance of timely PER degradation as a critical determinant in establishing 24-h rhythmicity. In addition to influencing protein degradation, DBT appears to affect the timing of nuclear accumulation of PER: The short-period mutant *dbt* ^S^ shows delayed PER nuclear accumulation that is apparently independent of PER protein stability [17], and arrhythmic alleles of *dbt* cause constitutive nuclear accumulation of PER in clock-containing cells of larval and adult *Drosophila* [18,19]. Lastly, in cultured cells reduction of DBT activity by RNA interference (RNAi) has been reported to reduce the potency of PER as a transcriptional repressor [20]. Thus, DBT appears to regulate different aspects of PER function in multiple steps throughout the circadian cycle.

The mutation *per* ^S^, which shortens period length to 19 h, was among the first circadian rhythm mutations isolated in *Drosophila* [21]. The mutation replaces serine with asparagine at amino acid (aa) position 589 (S589N) [22,23]. Similar, short-period behavioral phenotypes are produced by a wide variety of missense mutations affecting a region of approximately 20 aa encompassing the site of the *per* ^S^ mutation. The prevalence of such mutations suggests that short-period phenotypes may result from loss or depression of function in this region of the PER protein, which we will refer to as the “per-short domain” [24,25]. Importantly, the per-short domain has been implicated in the *trans*-regulation of DBT: mutations of this PER domain restore function to a kinase-deficient mutant, *dbt* ^AR^, suggesting that the wild-type form of the per-short domain may influence a temporal sequence of DBT-dependent phosphorylation(s) of PER [15].

In this paper, we describe genetic and biochemical approaches that have allowed the mapping of DBT-directed PER phosphorylation sites. These maps derive from interactions of *Drosophila* DBT and PER in vitro, and their functional significance has been explored in a cultured cell model and in living flies. Our studies have shown that the per-short domain and a neighboring segment of PER are preferentially phosphorylated by DBT. These adjoining domains appear to serve as alternative targets for DBT-directed phosphorylation. Interactions between these PER domains and DBT appear to provide a switch, giving either an unstable, highly active PER repressor, or a stable PER protein with little activity.

## Results

### Generation of Enzymatically Active DBT

**Figure 1:**
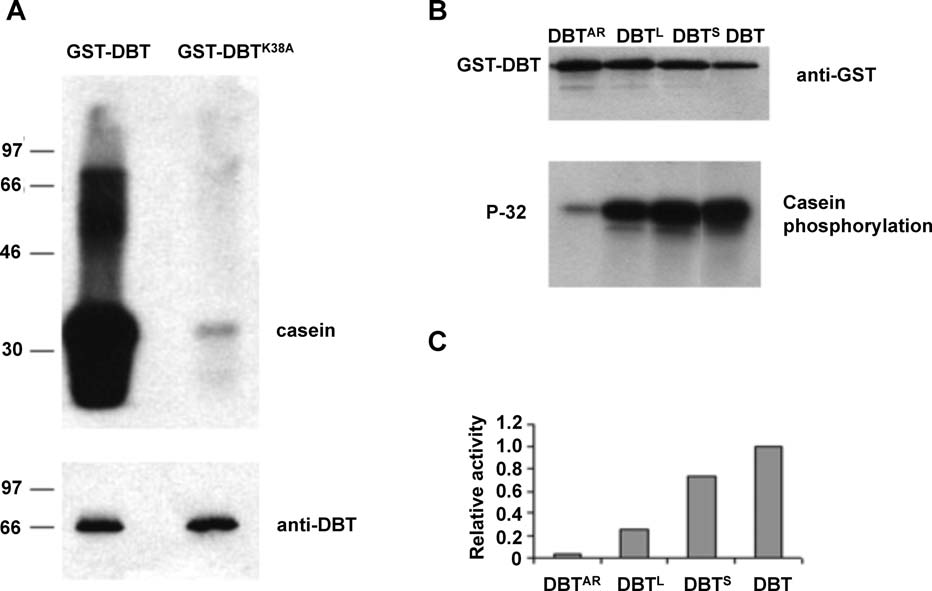
Activity of Recombinant DBT Purified from Sf9 Cells (A) Recombinant DBT purified from Sf9 cells has protein kinase activity. GST-DBT and GST-DBT (K38A) proteins purified from Sf9 cells were incubated with casein and radioactive P-32 labeled γ-ATP in kinase buffer. Casein is strongly phosphorylated by GST-DBT. Mutant GST-DBT (K38A) substantially reduces casein phosphorylation. The lower panel indicates the amount of GST-DBT and GST-DBT (K38A) used in the kinase assay as detected by western blot with anti-DBT antibody. Molecular weight markers are shown on the left. (B) DBT mutations affecting behavioral rhythms also affect kinase activity. Either wild-type DBT (DBT), DBT arrhythmic (DBT^AR^), long- (DBT^L^) or short- (DBT^S^) period rhythm mutants were expressed and purified as GST fusion proteins from Sf9 cells, and used to phosphorylate casein. Top: GST-DBT levels measured by anti-GST western blot. Bottom: equal amounts of casein were phosphorylated with wild-type GST-DBT (lane 4) or with mutant GST-DBT proteins: GST-DBT^AR^ (lane 1), GST-DBT^L^ (lane 2), and GST-DBT^S^ (lane 3). (C) Kinase activity of GST-DBT fusion proteins. Relative kinase activity is shown as a ratio of the casein phosphorylation signal to total GST-DBT level. Relative kinase activity of wild-type GST-DBT was set as 1.

Baculoviral expression in Sf9 cells was chosen for DBT production in an effort to recover kinase activity. Initially, efforts made to synthesize this *Drosophila* protein in bacterial cells (Escherichia coli) failed to produce enzymatically active DBT protein. Importantly, production of active mammalian CKIɛ using the same conditions in bacterial cells was successful, suggesting a specific problem emanating from the expressed DBT protein. Two baculoviruses were constructed. One expressed wild-type DBT, whereas a second generated a mutant form of DBT in which the putative ATP binding lysine (aa 38) was mutated to alanine (K38A). Several amino acid changes at this site have been previously shown to produce inactive CKI kinases [26]. Recombinant proteins were expressed as N-terminal glutathione S-transferase (GST)-fusion proteins to facilitate their purification. GST-DBT proteins were isolated 48 h after infection and initially tested for kinase activity using casein as an in vitro substrate. Whereas the fusion protein containing the wild-type DBT sequence produced strong phosphorylation of casein, the K38A mutant showed little or no activity (Figure 1A). This result demonstrates that DBT expression in Sf9 cells produces enzymatically active kinase and that the purified kinase activity is dependent on the DBT primary sequence. Recently, Muskus et al. [19] showed that a related (K38R) DBT mutant inhibits wild-type DBT function in a cultured cell phosphorylation assay and in behavioral tests of transgenic *Drosophila*.

### Enzymatic Activities of DBT Mutants Affecting Circadian Clock Function

Since DBT functions as a protein kinase, it is important to determine whether period-altering mutations that affect its structure also modify its biochemical activity. Single amino acid changes were introduced into the baculovirus construct, and mutant and wild-type kinases were purified in parallel. Thereafter, brief, timed kinase assays were performed using casein as a substrate (Materials and Methods). Each of the mutations decreased the kinase activity of DBT, but to varying degrees (Figure 1B). The most dramatic reduction was observed in the presence of the *dbt* ^AR^ mutation (Figure 1B, lane 1). When normalized to the kinase protein levels (Figure 1C), approximately 10% of the wild-type level of activity is preserved in DBT^AR^. The *dbt* ^L^ mutation reduced kinase activity to approximately 30% that of wild type. The *dbt* ^S^ mutation, which speeds up the clock in vivo, also reduced DBT activity by approximately 15% compared to wild type. The reduction of kinase activity by both period-shortening and lengthening mutations of *dbt* suggests complex regulation of the clock by DBT. Indeed, there is a precedent for such regulation in the case of a mammalian ortholog of DBT; the hamster CKI mutant *tau* speeds up the clock by promoting PER phosphorylations only at specific target sites and decreasing PER stability [27].

### DBT Phosphorylation of Clock Proteins

**Figure 2:**
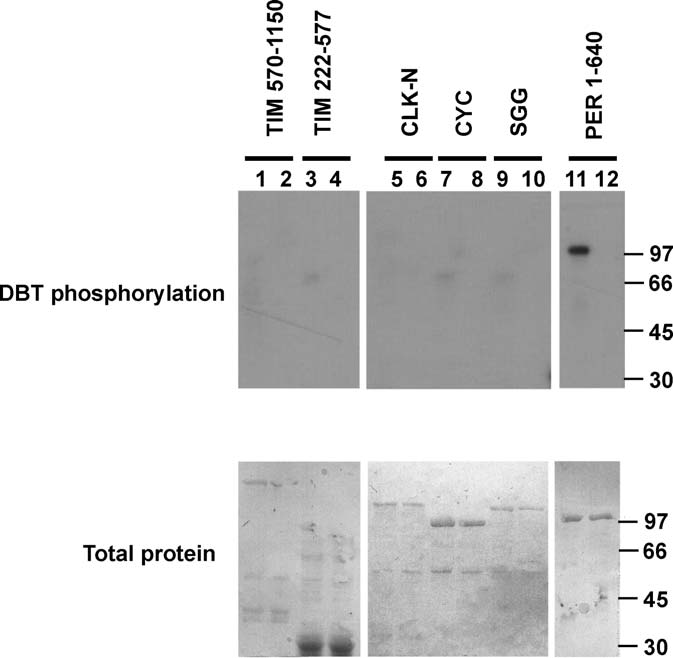
DBT Preferentially Phosphorylates PER Fragments or full-length sequences of proteins required for circadian clock function in *Drosophila* were fused to GST and expressed and purified from E. coli. Purified fusion proteins were treated with either wild-type DBT (odd-numbered lanes) or an inactive DBT mutant (K38A) as a negative control (even-numbered lanes). The top panel shows autoradiograph of incorporated phosphate; the lower panel shows total protein stained with Amido black. Molecular weight markers are indicated to the right of each panel. Sequences fused to GST as substrates for phosphorylation: lanes 1–2, TIM aa 507–1,150; lanes 3–4, TIM aa 222–577; lanes 5–6, CLK (-N terminus) aa 1–600; lanes 7–8, full-length CYC; lanes 9–10, full-length SGG; and lanes 11–12, PER amino acids 1–640. Molecular weight markers are shown on the right.

Availability of enzymatically active DBT allowed us to evaluate its action on clock-related substrates directly. Several clock proteins were expressed as GST fusions, and each was purified from E. coli and characterized with respect to its potential as a DBT substrate in vitro. Figure 2 shows that DBT specifically phosphorylated a GST-PER fusion protein encompassing the first 640 amino acids of *Drosophila* PER (Figure 2, lane 11). In contrast, no significant phosphorylation was observed for fusion proteins composing most of TIM, the N-terminal half of CLK, or full-length CYC and SGG (Figure 2). This experiment established that PER from *Drosophila* can be directly phosphorylated by the enzyme encoded by Drosophila dbt.

**Figure 3:**
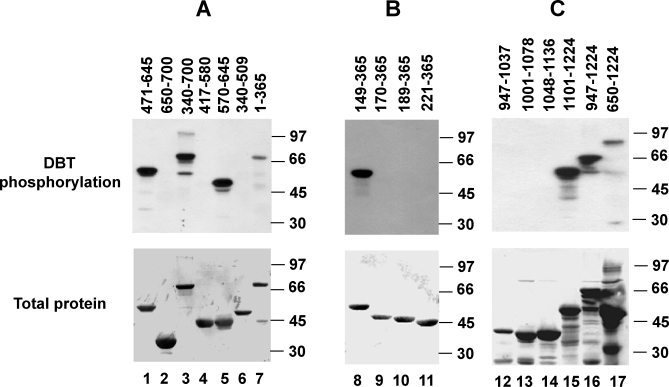
DBT Phosphorylates Three Regions in PER Different fragments of PER were fused to GST and expressed and purified from E. coli. Each fusion protein was phosphorylated with recombinant DBT, resolved in 10% SDS-PAGE, and transferred to nitrocellulose. Top panels in (A, (B), and (C) show signals from incorporated radioactive phosphate. Lower panels in (A), (B), and (C) show total proteins stained with Ponceau S. Numbers on top of each lane indicate the amino acids in PER included in each fusion protein. Molecular weight markers are shown on the right of each panel. (A) shows mapping of phosphorylation in the middle of PER (between residues 570–645). (B) shows mapping of phosphorylation near the N-terminus (between residues 149–170). (C) shows mapping of phosphorylation near the C-terminus (between residues 1,101–1,224).

Next, a more comprehensive mapping of the DBT-directed phosphorylation sites of PER was undertaken. A panel of overlapping GST fusion proteins was generated that together encompassed the full 1,224–amino acid sequence of *Drosophila* PER. Each fusion protein was allowed to react briefly with recombinant DBT in an effort to identify preferred DBT targets (Materials and Methods). Figure 3 shows patterns of phosphorylation for 17 PER fragments that define three regions of the protein that can serve as high-affinity in vitro targets for DBT.

PER 1–640, whose phosphorylation was initially observed in this way, was further subdivided to resolve two DBT target regions. A fusion protein containing the N-terminal 365 amino acids of PER was phosphorylated by DBT (Figure 3, lane 7), whereas a subfragment of this region, containing aa 170–365, was not (Figure 3, lane 9). In addition, PER 149–365 GST fusion protein showed a high level of phosphorylation (Figure 3, lane 8), indicating that the phosphorylated region closest to the N-terminus of PER is located between residues 149 and 170.

Sequences between aa 170 and 580 were not significantly phosphorylated by DBT (Figure 3, lanes 4, 6, and 9). Also, PER 650–700 was not phosphorylated by DBT (Figure 3, lane 2); however, strong phosphorylation of PER 570–645 was observed. Thus, a second phosphorylation region should reside between aa 580–645.

DBT also phosphorylated a fusion protein corresponding to the C-terminal half of PER (encoding residues 650–1,224) (Figure3, lane 17). Despite the presence of a multitude of C-terminal degradation products of this protein, only the highest mobility forms were significantly phosphorylated, suggesting that this DBT target occurs close to the C-terminus of PER. Shorter PER fragments collectively encoding residues 947–1,136 were not phosphorylated by DBT (Figure 3, lanes 12, 13, and 14). The strong phosphorylation of GST-PER 1,101–1,224 places the C-terminal DBT target within PER 1,136–1,224.

To summarize, in vitro DBT appears to preferentially phosphorylate sequences in three discrete regions of PER: aa 149–170, aa 580–645, and aa 1,136–1,224. All of these regions are serine- and threonine-rich (17%–26%). However, as further discussed below, a sequence motif previously implicated as a preferential target for phosphorylation in mammalian studies of CKI (S/TXXS/T) is encountered in only one of the target regions of PER. Although we have not determined whether the PER phosphorylation sites detected in vitro correspond to sites phosphorylated in vivo, the limited duration of the in vitro kinase assay is likely to favor phosphorylation of higher affinity sites, rather than secondary, nonspecific sites. Our analysis does not exclude the possibility that DBT phosphorylates PER at other sites in vivo.

### Identification of Individual DBT Phosphorylation Sites in PER

In order to define individual phosphorylation sites, three fusion proteins were chosen for phosphopeptide analyses. Fusion proteins (GST-PER149–365, GST-PER580–645, and GST-PER1101–1224) from each DBT target region were phosphorylated by recombinant DBT in the presence of radiolabeled ATP, and the resulting phosphorylated PER proteins isolated and subsequently digested with trypsin for further analysis (Materials and Methods). Table 1 shows the identified phosphopeptide sequences with phosphorylated residues indicated. The bulk of the radioactivity in the N-terminal PER target was located in one long peptide that contained the last 16 amino acids of GST followed by the first 27 amino acids of the fused PER sequence. The long N-terminal sequence composing the GST portion of the fusion protein precluded a further analysis by Edman sequencing. The recovery of this tryptic phosphopeptide (PER aa 149–176) was consistent with our earlier mapping of a preferential phosphorylation site(s) between aa 149 and aa 170. The amino acid sequence of the latter PER interval contains five serine residues at positions 149, 151, 153, 164, and 169. These residues are therefore potential substrates for DBT in the N-terminus of PER.

**Table 1:**
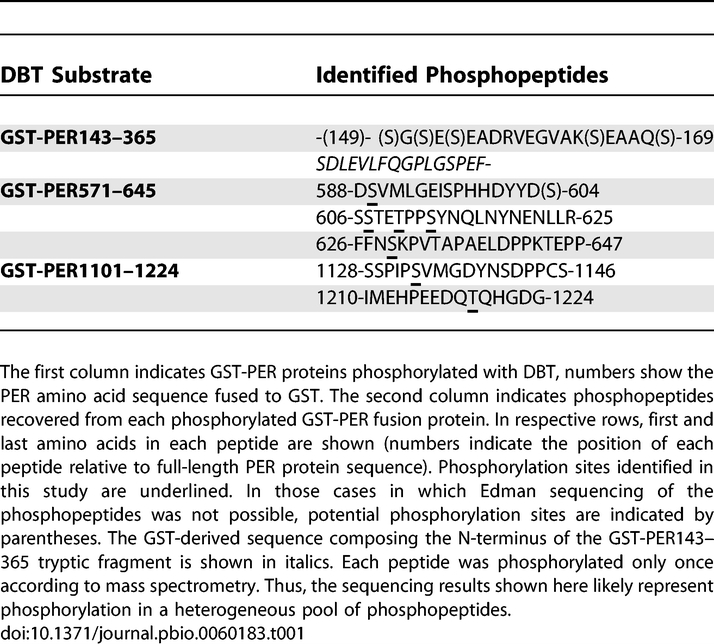
Phosphopeptides Generated from GST-PER Fusion Proteins by Reaction with DBT

Phosphorylation of GST-PER580–645 was predominantly associated with the three tryptic peptides shown in Table 1. The masses of each of these declined in response to alkaline phosphatase and propanethiol treatment (Materials and Methods). Amino terminal sequencing of these peptides, coupled with mass spectrometry, showed that the modified residues correspond to serines 589, 607, 613, and 629, and threonine 610. However, mass spectroscopy also indicated that none of the three peptides was detectably phosphorylated at multiple sites. Thus, each tryptic fraction proved to be a mixture of alternatively phosphorylated versions of the same peptide. Serine 604 was not amenable to Edman sequencing due to its position at the end of a longer tryptic peptide. This residue is part of a CKI consensus sequence (SKSS) that also includes serine 607, which was shown to be phosphorylated as indicated above.

**Figure 4:**
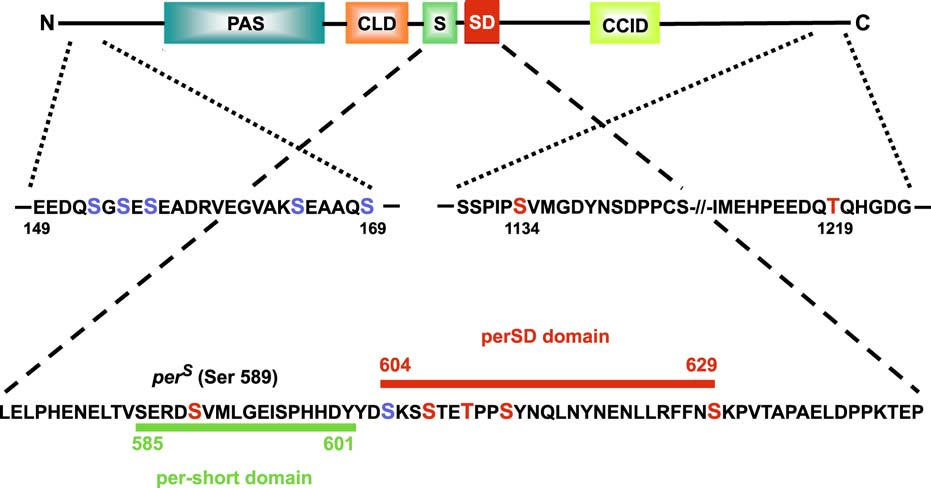
Distribution of Phosphorylated Regions in PER Relative to Functional Domains or Motifs Colored blocks show functional domains found in PER. Three regions phosphorylated by DBT are shown as segments of PER protein sequence, with phosphorylated residues established in this study indicated in red. Four serines between aa 143–169, as well as serine 604, are shown in blue, as they are potential targets within DBT-derived phosphopeptides, but could not be directly assessed for phosphorylation in this study; see also the text). PAS indicates position of PAS domain (contains aa ∼220–450) [51]; CLD, cytoplasmic localization domain that promotes PER cytoplasmic localization in the absence of TIM (aa 452–512) [52]; S, per-short domain, causes short-period behavioral rhythms when variably mutated (indicated as a green bar; 585 and 601 indicate the first and last amino acid of the motif, respectively) [24]; SD, per-short downstream domain, contains many DBT phosphorylation sites (indicated by the red bar above the protein sequence; 604 and 629 indicate the first and last amino acids, respectively, of the region examined in this study); CCID, CLK-CYC inhibitory domain, represses CLK-dependent transactivation when overexpressed in cultured cells [39]. (N and C indicate the amino- and carboxy-terminal ends of the schematic PER).

Two phosphopeptides were derived from the C-terminal fusion protein GST-PER1101–1224. Sequencing and mass spectrometry of these revealed one phosphorylated residue in each peptide, corresponding to serine 1,134 and threonine 1,219. Figure 4 shows a schematic representation of the identified DBT phosphorylation sites in PER in relation to previously recognized functional domains.

### Analysis of PERIOD Phosphorylation Site Mutants in Cultured S2 Cells

**Figure 5:**
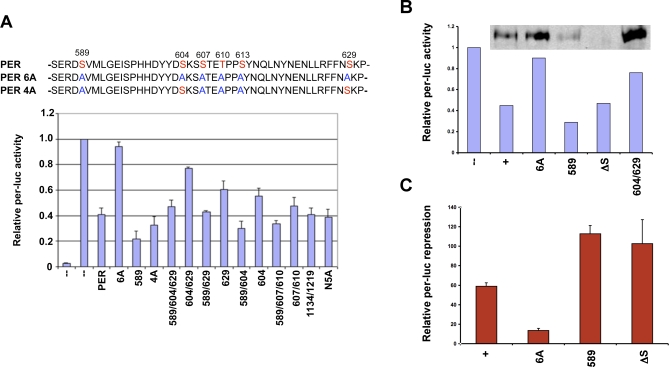
Repression of CLK-Dependent Activation of *per-luciferase* Reporter by PER Phosphorylation Site Mutants Bar graphs indicate relative luciferase activity levels from S2 cells cotransfected with *Clk* and 50 ng of either wild-type (+) or mutant *per* expression plasmids along with the *per-luc* reporter. Normalized luciferase activities were plotted as relative activation compared to expression in the absence of *per* (–). Numbers below the graph indicate positions of serine or threonine residues in PER that were mutated to alanine (PER mutant N5A has amino acids 149, 151, 153, 164, and 169 mutated to alanine, and PERΔS has a 51-nt deletion in the per-short region as described in Materials and Methods). (A) Repression activity of PER mutants. Sequence on top shows the DBT-mediated phosphorylation sites in the per-short and perSD domains (in red), and the multiple residues mutated to alanine (in blue) for mutants 4A and 6A. Values are averaged over three experiments; error bars indicate standard error of the mean. Lane 1, no added CLK. Remaining lanes, CLK plus indicated per PER repressor. (B) Stability of the PER mutants. The assay was performed as described above with S2 cells transfected with equal amounts (50 ng) of mutant or wild-type *per* DNA. The panel at the top shows the amount of PER protein detected by western blot analysis at the time the luciferase assay was conducted. In this case, equivalent, small samples of the luciferase assays were subjected to SDS-PAGE, and the presence of PER protein was detected using anti-PER antibodies. (C) Quantification of *per-luc* repression by PER mutants as a function of the amount of PER protein. Increasing amounts of *per^+^* and *per* mutant DNA were used to transfect S2 cells. In three independent experiments, the level of repression was determined as described above, and the amount of PER present in each assay quantified by western blot. The levels of repression shown represent activity per unit protein as assessed by the comparison of the quantified western blots. This comparison indicates that PER S589A and PERΔS are both more active repressors than the wild-type PER protein.

To examine the relevance of each DBT-derived phosphorylation of PER, site-directed mutagenesis of a *per* cDNA was performed. Full-length mutant proteins were subsequently expressed under control of an actin promoter and tested for their relative activity as transcriptional repressors in Schneider line 2 (S2) cultured *Drosophila* cells. This cultured cell assay measures CLK-dependent activation of the *per* promoter, comparatively, in the absence and presence of PER. A standard luciferase assay is employed to measure the effect of PER on CLK activity, so that relative luciferase levels that are expressed under the control of the *per* promoter report the CLK-dependent activation of transcription (cf. [20,28]). Selected sites were mutated individually, and some sites were also mutated combinatorially to investigate possible interactions between different phosphorylation targets. In all cases, each substitution was to alanine. Thirteen PER mutants were tested in this fashion, with the results shown in Figure 5A.

Mutations of the N-terminal and C-terminal PER phosphorylation sites had no detectable effect on PER's activity as a repressor of CLK (compare mutants N5A and 1,134/1,219 to wild-type PER in Figure 5A). In contrast, all mutations in the per-short downstream (perSD, Figure4) domain reduced PER function in this assay, and a mutation of serine 589, in the per-short domain, enhanced PER's activity as a CLK repressor (Figure5A). With respect to mutations of the perSD domain, the inhibitory effect varied depending on the residue(s) mutated: Strongest effects were seen in combinatorial mutations. For example, when residues 604 and 629 were both mutated to alanine, PER function was disabled to a greater extent than that of either single mutant (compare mutant 604/629 with 604 and 629 in Figure 5A). Little or no repressor activity was observed for a combinatorial PER mutant containing alanine substitutions at all per-short and perSD phosphorylation sites (mutant 6A in Figure 5A).

An inverse relationship was observed between S589A and mutations of the perSD domain. These combinations always showed intermediate levels of PER activity that suggested a partial restoration of function by S589A (compare mutant 589/604/629 to 604/629 and 589; 589/629 to 629 and 589; 589/604 to 604 and 589; and 589/607/610 to 607/610 and 589 in Figure 5A).

We further tested in the repression assay, a deletion of *per* that removes the complete per-short domain ([24], see also “*per*Δ*S* transgenes” below). Following transfection of S2 cells with equal amounts of DNA, both PER 589 and PER ΔS appeared to produce greater transcriptional repression than wild-type PER, despite the low levels of PER 589 and PER ΔS protein detected in these assays (see Figure 5B). In contrast, high levels of the PER 6A and PER 604/629 proteins produced only weak repression, suggesting that increased or decreased protein levels are not responsible for the apparent changes in activity levels. To further address this issue, we determined the level of repression associated with wild-type and mutant forms of PER as a function of the amount of protein present in the assay (see Figure 5C). When normalized to the wild-type PER protein levels, measured activities of the PER S589A and PERΔS repressors were higher than that of wild-type PER, indicating approximately twice the activity per unit protein. Together these S2 cell findings suggest that the PER-short domain regulates PER stability and PER's activity as a CLK repressor.

The functional analysis of phosphorylation sites presented here indicates that DBT-target residues in the per-short and perSD domains are relevant for PER-dependent repression of CLK. The amino acids phosphorylated by DBT in the N- and C-terminus did not have an effect in this assay when mutated, but may be relevant for aspect(s) of PER regulation that are not revealed in the S2 transcription assay.

### *per*Δ*S* Transgenes Dominantly Shorten the Period of Locomotor Rhythms

Serine 589 is mutated in the short-period (19-h) mutant *per* ^S^ [22,23], indicating its relevance to the regulation of *Drosophila*'s circadian rhythms [21]. Serine 589 is included in the per-short domain (Figure 4), a 17–amino acid interval, aa 585–601, throughout which single amino acid substitutions predominantly generate short-period phenotypes [24,25,29]. The latter finding suggests that short-period mutations cause a domain-specific loss of PER function [24]. To further test this hypothesis, a transgenic *Drosophila* strain was constructed with an intragenic PER deletion, termed *per*Δ*S*, affecting the entire per-short domain. The *per*Δ*S* transgene contained the complete sequence encoding PER with a 51-nucleotide (nt) in-frame deletion removing the DNA sequence encoding aa 585–601, thus also lacking serine 589, one of the identified DBT target sites. Further transgenic lines were created by genetic recombination. These carried one or two *per*Δ*S* transgenes in homozygotes (giving two and four copies of the transgene, respectively), or were heterozygous for one transgene. The recombinant flies were alternatively generated in wild-type or *per* null mutant backgrounds, and tested for locomotor activity in constant darkness. Table 2 shows the results of these behavioral assays (examples of individual locomotor activity records are presented in Figure S1). A progressive shortening of period length, and eventually arrhythmia, was observed as the number of *per*Δ*S* transgenes was increased. It has been suggested that mutations in the per-short domain generate short-period phenotypes through a mechanism that involves premature degradation of nuclear PER proteins [30]. However, if this were the only mechanism responsible for decreased period length in such mutants, it might be expected that progressively longer periods would result from increasing *per*Δ*S* copy number, in contrast to our observations. Our results suggest that a qualitative change in PER function contributes to these short period phenotypes.

**Table 2:**
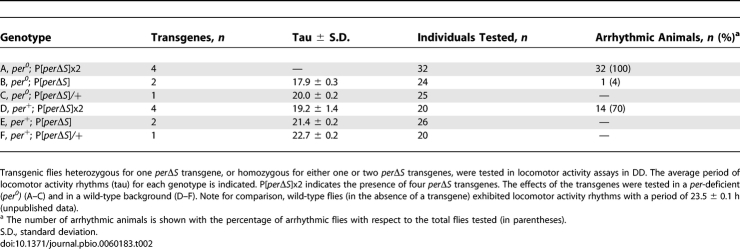
Dosage-Dependent Shortening of the Period Length of Locomotor Activity Rhythms by *per*Δ*S* Transgenes

### *Clock* Gene Expression in *per*Δ*S Drosophila*

To better understand the basis of the short period behavior of *per*Δ*S* flies, we examined the expression patterns of *Clk*, *tim*, *vri*, and *pdp1*. In each of these studies, *per* expression was monitored by RNase protection with a probe spanning the per-short domain allowing independent measurement of mRNA expression from the endogenous *per* gene and the *per*Δ*S* transgene(s).

**Figure 6:**
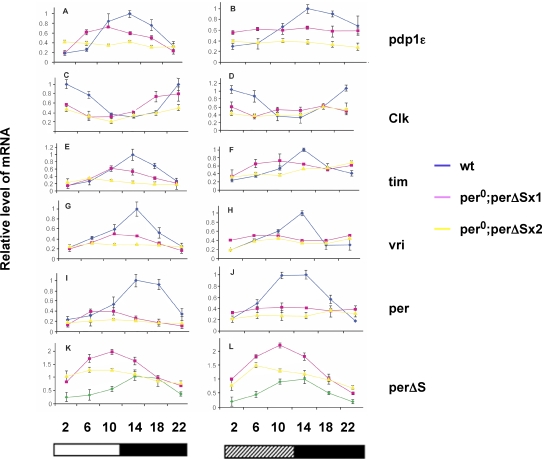
Quantification of Clock Gene mRNA Expression mRNA expression of cycling genes making up the circadian clock in flies was measured by northern blot or RNase protection from two time-course experiments (one of which is shown in Figure S2). An average of the normalized values was obtained and plotted. Genes assayed are indicated to the right of their respective profiles. RNA profiles on the left are from 12 h/12 h, light/dark cycles (LD, indicated by alternating open and closed horizontal bars), right-side profiles are from DD time courses (constant darkness with subjective day indicated by hatched bar). Numbers on the vertical axes indicate relative expression (maximal expression in wild type is 1.0). Numbers on the horizontal axes indicate time (hour within a 24-h cycle) when RNA was collected. Blue, wild-type (wt) time courses; pink, time courses from flies homozygous for one *per*Δ*S* transgene (*per^0^;per*Δ*Sx1*); and yellow, time courses from flies homozygous for two *per*Δ*S* transgenes (*per^0^;per*Δ*Sx2*). For (K) and (L), the green line represents the endogenous *per* RNA present in the *per^0^* background, to which the levels were normalized. Notice the change in the scale due to the increased expression of *per*Δ*S* RNA. Error bars show square root of variance from two independent samplings.

The combined results of two independent time-course studies are shown in each of the panels composing Figure 6. A representative mRNA expression profile from one of these time courses is shown in Figure S2. Lower levels of expression were observed in the transgenic animals for all genes monitored, and the amplitude of residual RNA rhythmicity was reduced. Moreover, stronger effects were observed as the number of transgenes increased, consistent with increased frequency of behavioral arrhythmia in these animals (Table 2).

In wild-type flies, the expression of *per*, *tim*, *vri*, and *pdp1*ɛ peaks during the early evening (approximately Zeitgeber time [ZT] 14; see wild type in Figure 6I, 6E, 6G, and 6A, respectively). In contrast, expression of these genes in flies with one homozygous *per*Δ*S* transgene showed an advanced phase, with peak expression time occurring in the afternoon (∼ZT 10; Figure 6I, 6E, 6G, and 6A). In each case, a lower amplitude RNA rhythm accompanied the advance phase of RNA expression. Corresponding alterations were observed in the *Clk* mRNA accumulation profile. In wild-type flies, *Clk* expression oscillates with a phase opposite to that of *per*, *tim*, *vri*, and *pdp1*ɛ. The phase of *Clk* RNA cycling is also advanced in flies carrying one homozygous *per*Δ*S* transgene (Figure 6C).

Expression levels of the *per*Δ*S* transcript were also measured. *per*Δ*S* mRNA showed oscillatory expression in animals with one and two homozygous *per*Δ*S* transgenes, with overall high levels of expression (Figure 6K and 6L). The observed increase in mRNA expression from *per*Δ*S* may be caused by an absence of dosage compensation that would regulate the endogenous *per* locus on chromosome X (both transgenes are in Chromosome 3). In fact, trough levels of *per*Δ*S* expression were comparable to peak expression levels of endogenous *per* mRNA in wild-type flies (Figure 6K and 6L).

### PER Protein Expression in *per*Δ*S* Flies

**Figure 7:**
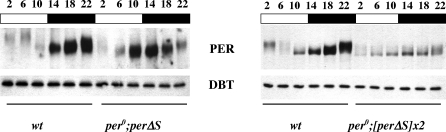
PERΔS Protein Expression in Flies with Two to Four *per*Δ*S* Transgenes Western blot analyses of PER from fly heads collected at the indicated times in LD (indicated by altering open and closed horizontal bars). Newly eclosed flies were entrained in LD for at least 3 d prior to collection. Genotypes assayed are indicated at the bottom of each panel (wt, wild type; *[per*Δ*S]x2* indicates presence of two homozygous *per*Δ*S* transgenes (four copies total). PERΔS protein signals are visible in the upper panels. Only PERΔS proteins were detected in transgenics due to the *per* null (*per* ^0^) background. DBT expression (lower panel) was used as a loading control.

The reduced expression of *per*, *tim*, *vri*, and *pdp1*ɛ observed in all *per*Δ*S* flies indicated that PER proteins missing the per-short domain are enhanced repressors of CLK, a result consistent with the behavior of per-short domain mutations in our cell culture assays of repressor activity (Figure 5). To determine whether elevated PERΔS protein accumulation is associated with the heightened suppression of CLK, anti-PER western blots were performed using flies with *per*Δ*S* transgenes in a *per* null background. In flies with one homozygous *per*Δ*S* transgene, PERΔS proteins were expressed rhythmically in light/dark (LD) cycles (Figure 7) and also in dark/dark (DD; unpublished data). PERΔS peak levels were advanced in these flies by 4 h when compared to PER accumulation profiles in wild-type flies (Figure 7). However, the abundance of PERΔS did not significantly differ from that seen for PER in wild-type flies. This was unexpected since a high level of *per*Δ*S* mRNA expression was found in these flies. The lack of protein overexpression in the presence of elevated mRNA levels is likely to indicate that PERΔS proteins are not as stable as wild-type PER proteins.

In the lines carrying two homozygous *per*Δ*S* transgenes, PERΔS protein levels were further reduced (Figure 7). Remarkably, PERΔS proteins accumulated with a modest rhythm despite the behavioral arrhythmicity of these flies. However, the electrophoretic mobility of PERΔS, which by comparison to wild-type PER should reflect the changing phosphorylation states of the protein during a 24-h cycle [31,32], was substantially altered such that chiefly hypophosphorylated protein accumulated throughout the cycle (Figure 7). As prior work has indicated that per-short domain mutations enhance DBT activity [15], and we have shown in this study that *per*Δ*S* flies produce elevated levels of *per*Δ*S* RNA, our results could indicate that PERΔS proteins are more efficiently phosphorylated and degraded than their wild-type counterparts. Consistent with this view, previous work has shown that in two mutants of the per-short domain, *per* ^S^ and *per* ^T^, hyperphosphorylated PER protein levels decline prematurely during the early night [30], and a reduction of DBT activity is associated with hypophosphorylation and stabilization of wild-type PER (cf. [12,15]).

## Discussion

Initial studies of *dbt* mutations suggested that PER is the primary target of DBT in the *Drosophila* circadian clock. Loss of *dbt* expression led to hypophosphorylation and overaccumulation of PER in vivo [12]. Mutations of *dbt* that shorten or lengthen behavioral rhythms led to correspondingly earlier or later degradation of PER in fly heads [12]. The sequence of *dbt* suggested that it encodes an ortholog of the CKI family of protein kinases in mammals [9]. Direct evidence for DBT kinase activity and PER phosphorylation has been hampered by the absence of specific enzymatic activity in bacterially produced recombinant DBT preparations [10,33], even though highly homologous mammalian CKIɛ (86% primary sequence identity in the kinase domain) has been recovered in an active form from bacterial expression systems. It is possible that for *Drosophila* DBT, protein folding is more sensitive to the cellular environment than the mammalian enzyme. Alternatively, DBT may require posttranslational modification(s) and/or the presence of a stimulating activity or cofactor(s) to produce an active conformation that is not available in bacterial cells. Nevertheless, in this report, we have shown that recombinant DBT can be expressed and recovered in an enzymatically active state from an insect cell line. However, even this insect-derived activity is short-lived (S. Kivimäe, unpublished data).

Recombinant DBT in vitro preferentially phosphorylates PER protein. No significant phosphorylation was observed when TIM, SGG, and CYC were used as a substrate in vitro. Although no direct DBT phosphorylation of the N-terminal half of CLK was observed, recent studies by others have shown that DBT is required for the generation of a highly phosphorylated and unstable form of CLK in vivo [4,34]. Furthermore, to achieve this highly phosphorylated state of CLK, it has been suggested that PER must be present and function as a bridge to physically deliver DBT to CLK [4]. CLK appears to be present in flies in multiple isoforms, including hypophosphorylated, intermediate-, and highly phosphorylated states. The last form is only observed during the late part of the CLK accumulation cycle and may promote CLK degradation [34]. The lack of in vitro phosphorylation of CLK by DBT in our current studies may reflect a DBT phosphorylation target in the C-terminal half of CLK, or a requirement for additional kinases to “prime” a DBT target, or a dependence on PER for DBT-directed phosphorylation of CLK.

Casein and PER are phosphorylated by enzymatically active recombinant DBT, and mutations of *dbt* that are known to affect the behavioral rhythm also have an effect on kinase activity. The short- and long-period mutations *dbt* ^S^ and *dbt* ^L^ [12] both reduce the kinase activity of DBT. Importantly, when the *dbt* ^S^ mutation was introduced specifically into the *Drosophila* kinase, the effect was far less severe than a previously reported orthologous substitution; using casein as a substrate, the mutation produced a 15% decrease in activity when composing the fly kinase (this study), but decreased enzyme activity by approximately 55% when introduced into *Xenopus* CKIδ [35]. Vertebrate CKI and DBT include divergent C-terminal protein segments, and the former enzyme cannot restore DBT function in transgenic *Drosophila* [36]. These results suggest that any analysis of DBT mutations in a heterologous system should be applied with caution. The comparatively subtle effect of *dbt* ^S^ on DBT activity versus its strong effect on period length could reflect a qualitative change in the mutant enzyme's activity. An assessment of potential differences of this sort might come from further mapping studies of the target specificities of DBT^S^ versus DBT on PER substrates in vitro.

Three distinct regions of PER are preferentially phosphorylated by DBT in vitro. In the C-terminal region of PER, we have identified two phosphorylation targets, serine 1,134 and threonine 1,219. No prior genetic or biochemical studies have suggested a specific role for this PER interval, and our cultured cell assays indicate that both phosphorylation targets can be removed without a detectable effect on PER's function as a CLK repressor (Figure 5). Near the N-terminus of PER, a region of 20 amino acids (residues 149–169) contains five serines that are candidate phosphorylation sites for DBT. This interval is located upstream of the PAS domain of PER. A region important for DBT/PER binding in vitro has been mapped to the first 300 amino acids of PER [9]. However, we have not determined whether there is a closer physical correspondence of the binding and phosphorylation target sequences, or whether elimination of potential DBT targets in this PER interval affects PER/DBT binding. As in the case of our cultured cell studies of serine 1,134 and threonine 1,219, elimination of all phosphorylation targets between aa 149–169, produced no detectable effect on PER-dependent repression in cultured cells.

Interestingly, two of the putative CKI sites, serine 151 and serine 153, are substrates of casein kinase 2 [37]. Transgenic flies carrying S151A and/or S153A mutations showed period lengthening and delayed PER nuclear localization. It will be important in future studies to distinguish CK2 and DBT phosphorylation targets in this region of PER and to determine their individual and/or interactive effects on PER function in vivo.

Of most interest in this study are phosphorylation sites identified in the third DBT target region of PER, between residues 580–645. This is a genetically well-studied interval previously implicated in the regulation of behavioral rhythms. As described earlier, mutations of a portion of this region, the per-short domain (Figure 4), are predominantly associated with short-period behavior [24,25,29]. The first mutation to be mapped to this region, *per* ^S^, [21,22,23] eliminates a candidate phosphorylation site at serine 589, and our S2 cell assays indicate that blocking phosphorylation of this site enhances PER's activity as a repressor of CLK (Figure 5). Earlier work has also shown that *per* ^S^ leads to premature nuclear degradation of PER [30], and our current studies of transgenic *Drosophila* producing a PER protein truncated by 17 amino acids including serine 589 (*per*Δ*S*), similarly indicate a role for this sequence in regulating PER stability and function as a repressor (Figures 6 and 7). Thus, the wild-type per-short domain appears to promote PER stability while reducing its activity as a transcriptional repressor.

**Figure 8:**
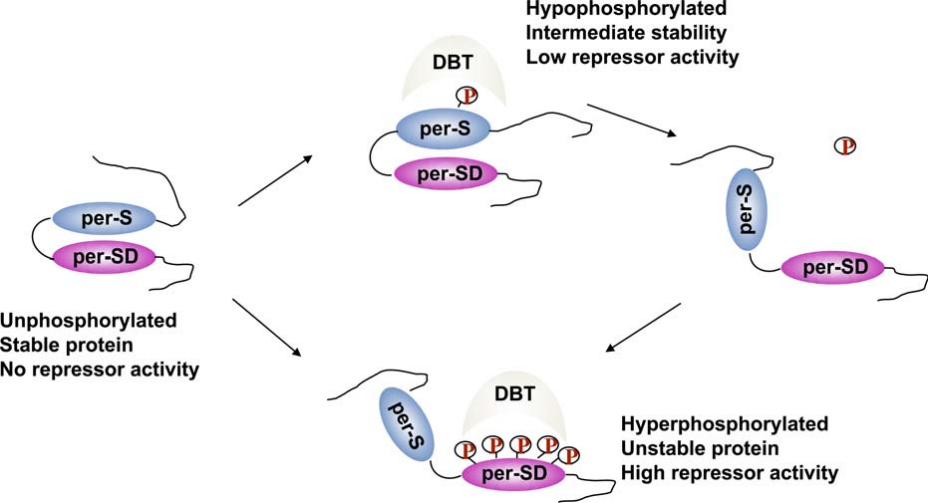
Activity of the *per*-Short and perSD Domains Regulated by DBT Phosphorylation A schematic of *Drosophila* PER protein is shown with the per-short (per-S) and per-short downstream (per-SD) domains highlighted. Residues phosphorylated by DBT in vitro are indicated (P). Three phosphorylation states are indicated, as well as effects on stability and activity as suggested by our studies of cultured cells and transgenic flies. It is proposed that phosphorylation of the *per*-short domain inhibits DBT-directed phosphorylation of the *per*-short downstream domain (see text).

We note that earlier work has also suggested that the mutation *per* ^S^ enhances DBT activity [15]. If serine 589 is no longer an available DBT target in *per* ^S^, by what mechanism might DBT function be elevated? Although mutating serine 589 enhances PER's activity as a repressor, this response is partially suppressed by a further mutation of some phosphorylation sites in the perSD region (Figure 5). As well, little or no repressor activity is detected if all phosphorylation targets are removed from perSD, even in the presence of the *per* ^S^ mutation (Figure 5). Together, these results suggest that the phosphorylation state of serine 589 may influence DBT activity on downstream targets within perSD that are required for PER's function as a CLK repressor. In such a model (Figure 8), dephosphorylation of the per-short domain would promote DBT-directed phosphorylation of perSD, enhancing PER's activity as a repressor and also destabilizing the protein. Reciprocally, phosphorylation of serine 589 would depress activity of DBT with respect to perSD, providing a more stable, but less active, PER protein. In this view, a fully dephosphorylated state would provide the most stable protein, but would provide an inactive form of PER, consistent with our findings in cultured cells (Figure 5) and transgenic flies (Figure 7), and with earlier studies of DBT-deficient *Drosophila* [12,15]. Further in vivo evidence comes from transgenic flies expressing PER that contains a deletion of a conserved segment (aa 516–568) downstream of the PAS domain [38]. This mutant PER protein displays increased stability and low-amplitude oscillations. More importantly, high levels of a hypophosphorylated form of PER are produced by these truncated proteins, which constitutively accumulate in the nucleus. This protein appears to have little or no activity as a transcriptional repressor [38].

Earlier studies of PER repression in S2 cells identified a region of PER, distinct from those reported here, that is also required for PER's function as a repressor of CLK-mediated transcription (CLK-CYC inhibition domain [CCID], aa 764–1,034; [39]). A novel, bipartite nuclear localization signal (NLS) is found in this region that influences nuclear localization of PER in S2 cells. Recent mapping studies of CCID led to the identification of a smaller, PER-DBT binding domain (PDBD; aa 755–809) that upon deletion increases PER stability, decreases PER phosphorylation, and severely impairs PER function as a transcriptional repressor [40]. A related deletion of PER (aa 768–792) produced similar defects, and impaired nuclear accumulation of PER in cultured cells [41]. Studies of the latter deletion in transgenic flies showed that a hypophosphorylated form of PER is constitutively expressed at high levels and is a very poor repressor. However, repressor function was restored by NLS addition, suggesting that dysfunction of the protein was likely due to its inability to localize to the nucleus [41].

The arrangement of four of the phosphorylation targets included within the perSD domain (serines 604, 607, and 613, and threonine 610) resembles a CKI phosphorylation motif found in human PER2 that has been implicated in FASPS. In the human PER protein, there are five serines spaced in an arrangement such that every third amino acid residue is a serine. These five serines are thought to be progressively phosphorylated by CKIɛ/δ, and in certain FASPS subjects, the first serine is mutated to glycine [42]. It has been proposed that this mutation inhibits the phosphorylation of the remaining serines, thus leading to altered hPER2 levels or activity in FASPS subjects [42]. In fact, more-recent studies of this mutation indicate that it may alter PER2′s repressor function because lower levels of *mper2* RNA are found in transgenic mice expressing the similarly mutated mPER2 protein [43]. Cultured cell studies have also suggested that the mutation affects mPER2 stability [44]. Accordingly, a role for CKI-directed phosphorylation in the regulation of PER's stability and activity as a transcriptional repressor may be conserved from flies to mammals.

## Materials and Methods

### Maintenance of cultured cells.

Sf9 cells (Invitrogen) were maintained at 28 °C in serum-free medium Sf-900 II SFM according to the supplier's recommendation. A new batch of cells was used every 3–4 mo. S2 cells were maintained at room temperature in *Drosophila* SFM medium (Invitrogen) supplemented with 15% fetal bovine serum and 1.5 g l-glutamine/500 ml.

### Cloning of plasmid constructs.

PER coding sequences were amplified by PCR and cloned between EcoRI and XhoI sites in a pGEX6P-1 expression plasmid. For GST-SGG and GST-CYC, full-length open reading frames of *sgg* and *Bmal* were amplified by PCR and cloned into pGEX-6P-1 between SmaI and NotI sites and EcoRI and XhoI sites, respectively. For GST-DBT, the *dbt* open reading frame was PCR amplified and cloned as an EcoRI-XhoI fragment into pGEX-6P-1 vector. GST-TIM constructs have been described before [45].

For GST-DBT baculovirus construction, the GST-DBT coding sequence from pGEX-6P-1 vector was released as an SspI fragment and ligated in EcoRV site in the pFastBac-1 vector (Invitrogen). The resulting pFastBac-GST-DBT construct was used to transform DH10Bac competent cells (Invitrogen), and recombinant baculovirus DNA was selected and purified following the Bac-to-Bac system (Invitrogen) instructions. Baculovirus encoding for DBT mutants were constructed analogously, except point mutations changing the relevant amino acids were first introduced into the *dbt* coding sequence.

For S2 cell expression, *per* cDNA was cloned into a pAct plasmid. Point mutations into the *per* coding sequence were introduced by following the QuickChange II (Stratagene) mutagenesis protocol. Expression plasmids encoding for luciferase, *Clk*, and *lacZ* under actin promoter control have been described [46]. *per-luc* reporter construct contains a luciferase coding sequence under a *per* gene promoter cloned into a pCasper-4 plasmid [47].

### Expression and purification of recombinant PER proteins from *E. coli.*

Proteins were expressed in BL21pLysS cells (Novagen). Protein expression was induced with 1 mM IPTG at a cell density of optical density at 600 nm (OD_600_) = 0.6–0.8 for 3–4 h at 37 °C. After induction, cells were pelleted and resuspended in STE buffer (150 mM NaCl, 50 mM Tris [pH 8.0], 1 mM EDTA), and sarcosyl in STE was added to a final concentration of 1%. Resuspended cells were frozen once. Thawed lysate was passed through an 18-gauge syringe needle five times to fragment DNA. Shared lysate was centrifuged at 15,000*g* for 30 min at 4 °C. After centrifugation, Triton X-100 in STE was added to the supernatant to 2% final concentration and incubated with glutatione sepharose for 30 min at 4 °C. Bound protein was washed once with HND600 buffer (600 mM KCl, 20 mM Tris [pH 7.5], 5 mM EDTA, 10% glycerol, 0.5% Tween 20) and four times with HND100 (same as HND600 except with 100 mM KCl). Bound proteins were stored at −20 °C in 50% glycerol/HND600.

### Expression and purification of recombinant DBT proteins from Sf9 cells.

Sf9 cells were transfected with baculovirus DNA containing the GST-DBT expression cassette using Lipofectamine (Invitrogen) according to the manufacturer's recommendations. At 72 h after transfection, cell culture medium was collected, and transfected cells were lysed and tested for GST-DBT protein expression by western blot analysis. Anti-GST antibody (Santa Cruz Biotechnology) was used to detect recombinant protein expression. Cell culture medium from cells that tested positive for recombinant protein expression was used to infect cells for 48 h to produce GST-DBT baculovirus stocks. Viral stocks were stored at 4 °C. For protein expression, 0.3–1 ml of viral stock was used to infect approximately 1.5 × 10^7^ adherent cells in a 150-cm^2^ tissue culture flask in 25 ml of medium. Cells were lysed 48 h after infection in HND100 buffer on ice for 10 min in the presence of protease inhibitors III (Calbiochem). Lysates were cleared by centrifugation at 15,000*g* for 10 min. After centrifugation extracts were incubated shaking with glutathione sepharose at 4 °C for 30 min. Resin with bound protein was washed three times with lysis buffer and once with PreScission protease cleavage buffer (150 mM NaCl, 50 mM Tris [pH 7.5], 1 mM EDTA). Recombinant DBT protein was cleaved off the glutathione sepharose in overnight incubation with PreScission protease (Amersham) in 0.5 ml of protease buffer following the conditions recommended by the manufacturer. Next-day supernatants from protease cleavage were concentrated approximately 20-fold by ultrafiltration in Microcon centrifugal filters with molecular cutoff of 30 kD (Millipore). Resulting concentrates were used within hours for kinase assays (and contained ∼50 ng protein/μl). The activity was diminished to almost undetectable levels in 24 h after purification.

### In vitro protein phosphorylation.

GST-PER fusion proteins bound to glutathione sepharose beads were washed once with kinase buffer (50 mM Tris [pH 7.5], 10 mM MgCl_2_, 5 mM DTT) and resuspended in kinase buffer. A stock solution containing DBT protein purified from Sf9 cells was added (1/10 total reaction volume, 5 ng/μl final concentration) to resuspended beads after addition of 0.5 μl radioactive ATP labeled with P-32 at γ-phosphate (3,000 Ci/mmol) and 100 μM unlabeled ATP. Reactions were carried out in 30 μl and were allowed to proceed for 1 min at room temperature to reduce nonspecific phosphorylation. Reactions were stopped by adding equal volume of 2× SDS-PAGE loading buffer (125 mM Tris [pH 6.8], 4% SDS, 20% glycerol, 10% β-mercaptoethanol [v/v]). Phosphorylated proteins in SDS-PAGE loading buffer were boiled for 5 min and were resolved in 10% SDS-PAGE. Reactions with casein were carried out in similar conditions, except DBT proteins were not cleaved off the glutathione sepharose beads. A total of 100 ng of casein was used in each reaction.

### Phosphopeptide sequencing and mass spectrometry.

DBT-phosphorylated (^32^P) GST-PER fusion proteins were resolved in acrylamide gels and then stained and excised for subsequent analysis at the Rockefeller University Protein Research Facility according to facility procedures. Phosphorylated proteins were digested with trypsin, and the resulting peptides were resolved into approximately 100 fractions for each protein by reverse-phase high-performance liquid chromatography (HPLC) using a VYDAC C18 column (1.0-mm ID × 250-mm length) in a HPLC Agilent model 1100. Fractions that contained the majority of the radioactivity were treated individually with alkaline phosphatase and propanethiol (Figure S3). Treated fractions were analyzed by mass spectrometry, and peptide masses of treated and nontreated samples were compared. Alkaline phosphatase hydrolysis reduces peptide mass by 80 Da per phosphorylated residue. As an independent test, phosphopeptides were reacted with propanethiol. This results in a substitution reaction at the phosphorylation site and reduces peptide mass by 21 Da per phosphorylated residue. Peptide fractions that responded to both treatments were subjected to Edman sequencing to identify the position of individual phosphorylated amino acids in the peptides.

### Luciferase assay.

Luciferase assays were carried out essentially as described [45]. S2 cells were transfected in 24-well plates at 60%–80% confluency using Effectene transfection reagent (Qiagen) following the manufacturer's recommendations. Cells were transfected with 50-ng pAct-PER, encoding for either wild-type or mutant PER, 5-ng pAct-Clk, 10-ng per-luc, 20-ng pAct-lacZ, and 115 ng of pAct plasmid without an insert. Transcription in these assays depends on endogenously produced CYC and pAct-Clk–induced TIM (cf. [48]). Cells were harvested 24–36 h after transfection and lysed in Cell Culture Lysis Reagent (Promega). Extracts were mixed with Luciferase Assay Reagent (Promega) in a 1:5 ratio, and luciferase activity was measured using liquid scintillation counter LS6000IC (Beckman) in single-photon collection mode. Luciferase activity was normalized to cotransfected β-gal activity in each sample. To determine the amount of PER protein present in each luciferase assay, a small sample was subjected to SDS-PAGE, and the presence of PER protein was detected using anti-PER antibodies. To quantitate the strength of repression as a function of the amount of protein, S2 cells were transfected with increasing amounts of *per* DNA. Half of each cell sample was used to measure PER-dependent repression, with the remaining half used to determine the amount of PER protein in the assay.

### Protein extraction and western analysis.

Total protein from fly heads was extracted in HE buffer (20 mM Tris [pH 7.5], 100 mM KCl, 10 mM EDTA, 0.1% Triton, 1 mM DTT) supplemented with protease and phosphatase inhibitors (Calbiochem). Protein concentration was measured with BCA protein measurement kit (Pierce) according to the manufacturer's protocol.

For detection of PER and TIM proteins, 20 μg of total protein was resolved in 6% SDS-PAGE. The following antibodies at indicated dilutions in 5% nonfat milk were used for protein detection: anti-TIM 1:2,000 [49], anti-PER 1:20,000 (gift from Dr. J. Hall), or anti-PER 1:600 (Chemicon). Appropriate species-specific horseradish peroxidase–coupled secondary antibodies (Jackson Laboratories) were used at 1:10,000 dilution. Antibody binding was detected by ECL western blotting detection reagent (Amersham). Primary antibody incubations were carried out overnight at 4 °C. Secondary antibody incubations were carried out at room temperature for 1 h. Blots were washed with 1× TBST (50 mM Tris [pH 8.0], 150 mM NaCl, 0.5% Tween 20) three times for 10 min. Secondary antibody incubations were also carried out in 1× TBST.

### RNA isolation and northern analysis.

RNA was isolated from fly heads by homogenization in RNA STAT-60 extraction reagent (TEL-TEST) according to the manufacturer's protocol or with RNeasy RNA isolation kit (Qiagen) according to animal cell RNA isolation protocol with additional centrifugation after initial homogenization of fly heads and prior to column binding. Two micrograms to 20 μg of total RNA was resolved in 1% formaldehyde gel following the RNeasy Northern gel protocol and transferred to NytranN membrane (Schleicher and Schuell) in 10× SSC. Membranes were UV crosslinked in Stratalinker (Stratagene). Prehybridization was carried out for 0.5–2 h, and hybridization was performed overnight in 10 ml of Ultrahybe buffer (Ambion). Blots were washed four times in washing buffer (0.2× SSC, 1% SDS) at 65 °C. Signal was detected and quantified with Typhoon 9200 imager (Amersham).

Radioactive probes were generated using a Decaprime II (Ambion) DNA labeling kit according to the manufacturer's protocol. Full cDNA sequences were used to make probes for detection of *per*, *tim*, *vri*, *clk*, *dbt*, *cyc*, and *rp49* mRNAs. For pdp1ɛ mRNA detection, sequence specific for the ɛ isoform was used as a template for probe synthesis.

### RNase protection assay.

RNase protection assays were carried out using an RPAIII kit (Ambion) according to the manufacturer's protocol. For hybridization, 2–5 μg of total RNA from fly heads was used. For probe generation, a fragment of *per* genomic sequence containing the last 110 nt from intron 4 and the first 311 nt from exon 5 was amplified by PCR and cloned into pBluescript plasmid between EcoRI and Xho sites in the direction that allowed the antisense strand to be transcribed by T7 polymerase. This probe protects the 311 nt of *per* mRNA that includes the per-short domain. In *per*Δ*S* flies, the protected fragment is 51 nt shorter. Probe synthesis was carried out using a MaxiScript kit (Ambion) according to the manufacturer's protocol.

### Fly culture and locomotor behavior analysis.

Drosophila melanogaster were reared on standard medium at room temperature (∼25 °C). Monitoring and analysis of locomotor activity of individual flies was performed in constant darkness at 25 °C using the *Drosophila* Activity Monitoring System IV (Trikinetics).

### Generation of *per*Δ*S* transgenes.

Single-stranded DNA (ssDNA) site-directed mutagenesis with phosphorothioate incorporation [50] was used to delete the per-short region of the *per* gene. Briefly, a 30mer oligonucleotide, of which 15 nt mapped 5′ and 15 nt mapped 3′ of *per* deletion (indicated by “||” below), was synthesized (5′-AAC GAG TTG ACC GTC||TAT GAC AGT AAG AGT-3′). The oligonucleotide was phosphorylated with T4 polynucleotide kinase and annealed to the ssDNA XmaI-XmnI fragment recovered from *per* genomic DNA and cloned in pGEM 7-zf. The second strand was synthesized with DNA polymerase (Klenow fragment), and T4 DNA ligase was added to close the gap of the new strand. Nci I and exonuclease III were used to nick and then partially digest the parent strand. The mutant homoduplex was formed by incubating this partially double-stranded DNA in the presence of DNA polymerase I and T4 DNA ligase. PCR and DNA sequencing confirmed the deletion, and the fragment was cloned in the context of full-length genomic *per* DNA and pCasPer transformation vector. Transgenic flies carrying the *per*Δ*S* construct were generated and mapped by standard protocols.

## Supporting Information

Figure S1 Locomotor Activity of *per*Δ*S* flies Flies were entrained to an LD cycle for 3 d before testing. Thereafter, locomotor activity of individual flies was recorded in constant darkness at 25 °C. Representative actograms (top) and periodograms (bottom) for each genotype assayed are shown. For each periodogram, the lower limit of statistical significance (*p* < 0.01) is indicated by a slanted line.
(112 KB PDF)

Figure S2 mRNA Expression of Cycling Clock Genes in Flies Homozygous for One or Two *per*Δ*S* Transgenes RNA samples were collected for 1 d in LD (indicated by altering open and closed horizontal bars in (A), (C), and (E)), and 1 d in DD (subjective day indicated by hatched bars, in (B), (D), and (F)). Genotypes are shown at the bottom of each panel, and RNA profiles assessed are indicated on the left. Constitutive tubulin, rp49, and *cyc* mRNAs were measured for normalization. (E) and (F) are the results of an RNAase protection assay. Top, middle, and bottom bands in (E) and (F) represent protected fragment for tubulin, endogenous *per* gene mRNA, and protected fragment from transgenic *per*Δ*S*, respectively. Numbers indicate hours in a 24-h cycle when RNA was collected.
(715 KB PDF)

Figure S3 Mass Spectrometry of PERIOD Protein Phosphorylated by DBT Recombinant PER fragments were individually phosphorylated with DBT and digested with trypsin. (A) and (B) were derived from peptide 606-SSTETPPSYNQLNYNENLLR-625. Each peptide fraction containing the majority of the radioactivity was treated with propanethiol (A) or phosphatase (B) and analyzed by mass spectrometry. The peptide masses of untreated (top) and treated samples (bottom) were compared to identify the phosphorylated residue(s).
(A) shows an example of propanethiol treatment. Notice the change in the masses of the peptide 2422.29 after treatment due to a substitution reaction at the phosphorylation site, which reduces peptide mass by 21 Da per phosphorylated residue, improving ionization efficiency.
(B) shows the results obtained following alkaline phosphatase treatment, leading to a reduction of peptide mass by 80 Da per phosphorylated residue. Peptide fractions that responded to both treatments were subjected to Edman sequencing to identify the position of individual phosphorylated amino acids in the peptides.
(C) is a tandem mass spectrometry (MS/MS) spectrum of the PER peptide 1124-EVPDSSPIPSVMGDYNSD-1143, which is phosphorylated at position 10 (S-1134). The MS/MS spectrum shows a doubly charged ion at m/z 994.9 (Pre) corresponding to the mass of the phosphorylated peptide. A loss of 98 Da (H_3_PO_4_) was observed for this precursor and some fragment ions under low collision-induced dissociation. The peaks labeled y1–7 and b6–9 correspond to the masses of b and y fragment ions of unmodified peptides. The addition of 80 Da (mass of PO_3_) was observed for the b10 (b10+80) and y9 (y9+80) ions, demonstrating that phosphorylation was on the Ser at position 10.
(335 KB PDF)

## Abbreviations

aa: amino acid
*CKI*: casein kinase I
*Clk*: *Clock*
*cyc*: *cycle*
DD: dark/dark
FASPS: familial advanced sleep phase syndrome
GST: glutathione S-transferase
LD: light/dark
nt: nucleotide
*Pdp1*ɛ: *Par domain protein 1*ɛ
*per*: *period*
perSD: per-short downstream
SGG: Shaggy
*tim*: *timeless*
*vri*: *vrille*

## References

[pbio-0060183-b001] Allada R, Meissner RA (2005). Casein kinase 2, circadian clocks, and the flight from mutagenic light. Mol Cell Biochem.

[pbio-0060183-b002] Nakajima M, Imai K, Ito H, Nishiwaki T, Murayama Y (2005). Reconstitution of circadian oscillation of cyanobacterial KaiC phosphorylation in vitro. Science.

[pbio-0060183-b003] Hardin PE (2006). Essential and expendable features of the circadian timekeeping mechanism. Curr Opin Neurobiol.

[pbio-0060183-b004] Yu W, Zheng H, Houl JH, Dauwalder B, Hardin PE (2006). PER-dependent rhythms in CLK phosphorylation and E-box binding regulate circadian transcription. Genes Dev.

[pbio-0060183-b005] Cyran SA, Buchsbaum AM, Reddy KL, Lin MC, Glossop NR (2003). vrille, Pdp1, and dClock form a second feedback loop in the Drosophila circadian clock. Cell.

[pbio-0060183-b006] Benito J, Zheng H, Hardin PE (2007). PDP1epsilon functions downstream of the circadian oscillator to mediate behavioral rhythms. J Neurosci.

[pbio-0060183-b007] Xu Y, Padiath QS, Shapiro RE, Jones CR, Wu SC (2005). Functional consequences of a CKIdelta mutation causing familial advanced sleep phase syndrome. Nature.

[pbio-0060183-b008] Toh KL, Jones CR, He Y, Eide EJ, Hinz WA (2001). An hPer2 phosphorylation site mutation in Familial Advanced Sleep Phase syndrome. Science.

[pbio-0060183-b009] Kloss B, Price JL, Saez L, Blau J, Rothenfluh A (1998). The Drosophila clock gene double-time encodes a protein closely related to human casein kinase Iepsilon. Cell.

[pbio-0060183-b010] Kloss B, Rothenfluh A, Young MW, Saez L (2001). Phosphorylation of period is influenced by cycling physical associations of double-time, period, and timeless in the Drosophila clock. Neuron.

[pbio-0060183-b011] Zilian O, Frei E, Burke R, Brentrup D, Gutjahr T (1999). double-time is identical to discs overgrown, which is required for cell survival, proliferation and growth arrest in Drosophila imaginal discs. Development.

[pbio-0060183-b012] Price JL, Blau J, Rothenfluh A, Abodeely M, Kloss B (1998). double-time is a novel Drosophila clock gene that regulates PERIOD protein accumulation. Cell.

[pbio-0060183-b013] Ko HW, Jiang J, Edery I (2002). Role for Slimb in the degradation of Drosophila Period protein phosphorylated by Doubletime. Nature.

[pbio-0060183-b014] Deshaies RJ (1999). SCF and Cullin/Ring H2-based ubiquitin ligases. Annu Rev Cell Dev Biol.

[pbio-0060183-b015] Rothenfluh A, Abodeely M, Young MW (2000). Short-period mutations of per affect a double-time-dependent step in the Drosophila circadian clock. Curr Biol.

[pbio-0060183-b016] Kivimae S, Young MW, Saez L, Bradshaw RDE (2003). Casein kinase I and regulation of the circadian clock. Handbook of cell signaling.

[pbio-0060183-b017] Bao S, Rihel J, Bjes E, Fan JY, Price JL (2001). The Drosophila double-timeS mutation delays the nuclear accumulation of period protein and affects the feedback regulation of period mRNA. J Neurosci.

[pbio-0060183-b018] Cyran SA, Yiannoulos G, Buchsbaum AM, Saez L, Young MW (2005). The double-time protein kinase regulates the subcellular localization of the Drosophila clock protein period. J Neurosci.

[pbio-0060183-b019] Muskus MJ, Preuss F, Fan JY, Bjes ES, Price JL (2007). Drosophila DBT lacking protein kinase activity produces long-period and arrhythmic circadian behavioral and molecular rhythms. Mol Cell Biol.

[pbio-0060183-b020] Nawathean P, Rosbash M (2004). The doubletime and CKII kinases collaborate to potentiate Drosophila PER transcriptional repressor activity. Mol Cell.

[pbio-0060183-b021] Konopka RJ, Benzer S (1971). Clock mutants of Drosophila melanogaster. Proc Natl Acad Sci U S A.

[pbio-0060183-b022] Baylies MK, Bargiello TA, Jackson FR, Young MW (1987). Changes in abundance or structure of the per gene product can alter periodicity of the Drosophila clock. Nature.

[pbio-0060183-b023] Yu Q, Colot HV, Kyriacou CP, Hall JC, Rosbash M (1987). Behaviour modification by in vitro mutagenesis of a variable region within the period gene of Drosophila. Nature.

[pbio-0060183-b024] Baylies MK, Vosshall LB, Sehgal A, Young MW (1992). New short period mutations of the Drosophila clock gene per. Neuron.

[pbio-0060183-b025] Rutila JE, Edery I, Hall JC, Rosbash M (1992). The analysis of new short-period circadian rhythm mutants suggests features of D. melanogaster period gene function. J Neurogenet.

[pbio-0060183-b026] Longenecker KL, Roach PJ, Hurley TD (1996). Three-dimensional structure of mammalian casein kinase I: molecular basis for phosphate recognition. J Mol Biol.

[pbio-0060183-b027] Gallego M, Virshup DM (2007). Post-translational modifications regulate the ticking of the circadian clock. Nat Rev Mol Cell Biol.

[pbio-0060183-b028] Darlington TK, Wager-Smith K, Ceriani MF, Staknis D, Gekakis N (1998). Closing the circadian loop: CLOCK-induced transcription of its own inhibitors per and tim. Science.

[pbio-0060183-b029] Hamblen MJ, White NE, Emery PT, Kaiser K, Hall JC (1998). Molecular and behavioral analysis of four period mutants in Drosophila melanogaster encompassing extreme short, novel long, and unorthodox arrhythmic types. Genetics.

[pbio-0060183-b030] Marrus SB, Zeng H, Rosbash M (1996). Effect of constant light and circadian entrainment of perS flies: evidence for light-mediated delay of the negative feedback loop in Drosophila. EMBO J.

[pbio-0060183-b031] Edery I, Zwiebel LJ, Dembinska ME, Rosbash M (1994). Temporal phosphorylation of the Drosophila period protein. Proc Natl Acad Sci U S A.

[pbio-0060183-b032] Zeng H, Qian Z, Myers MP, Rosbash M (1996). A light-entrainment mechanism for the Drosophila circadian clock. Nature.

[pbio-0060183-b033] Suri V, Hall JC, Rosbash M (2000). Two novel doubletime mutants alter circadian properties and eliminate the delay between RNA and protein in Drosophila. J Neurosci.

[pbio-0060183-b034] Kim EY, Edery I (2006). Balance between DBT/CKIepsilon kinase and protein phosphatase activities regulate phosphorylation and stability of Drosophila CLOCK protein. Proc Natl Acad Sci U S A.

[pbio-0060183-b035] Preuss F, Fan JY, Kalive M, Bao S, Schuenemann E (2004). Drosophila doubletime mutations which either shorten or lengthen the period of circadian rhythms decrease the protein kinase activity of casein kinase I. Mol Cell Biol.

[pbio-0060183-b036] Sekine T, Yamaguchi T, Hamano K, Young MW, Shimoda M (2008). Casein kinase I epsilon does not rescue double-time function in Drosophila despite evolutionarily conserved roles in the circadian clock. J Biol Rhythms.

[pbio-0060183-b037] Lin JM, Kilman VL, Keegan K, Paddock B, Emery-Le M (2002). A role for casein kinase 2alpha in the Drosophila circadian clock. Nature.

[pbio-0060183-b038] Schotland P, Hunter-Ensor M, Lawrence T, Sehgal A (2000). Altered entrainment and feedback loop function effected by a mutant period protein. J Neurosci.

[pbio-0060183-b039] Chang DC, Reppert SM (2003). A novel C-terminal domain of drosophila PERIOD inhibits dCLOCK:CYCLE-mediated transcription. Curr Biol.

[pbio-0060183-b040] Kim EY, Ko HW, Yu W, Hardin PE, Edery I (2007). A DOUBLETIME kinase binding domain on the Drosophila PERIOD protein is essential for its hyperphosphorylation, transcriptional repression, and circadian clock function. Mol Cell Biol.

[pbio-0060183-b041] Nawathean P, Stoleru D, Rosbash M (2007). A small conserved domain of Drosophila PERIOD is important for circadian phosphorylation, nuclear localization, and transcriptional repressor activity. Mol Cell Biol.

[pbio-0060183-b042] Toh KL, Jones CR, He Y, Eide EJ, Hinz WA (2001). An hPer2 phosphorylation site mutation in familial advanced sleep phase syndrome. Science.

[pbio-0060183-b043] Xu Y, Toh KL, Jones CR, Shin JY, Fu YH (2007). Modeling of a human circadian mutation yields insights into clock regulation by PER2. Cell.

[pbio-0060183-b044] Vanselow K, Vanselow JT, Westermark PO, Reischl S, Maier B (2006). Differential effects of PER2 phosphorylation: molecular basis for the human familial advanced sleep phase syndrome (FASPS). Genes Dev.

[pbio-0060183-b045] Martinek S, Inonog S, Manoukian AS, Young MW (2001). A role for the segment polarity gene shaggy/GSK-3 in the Drosophila circadian clock. Cell.

[pbio-0060183-b046] Rothenfluh A, Young MW, Saez L (2000). A TIMELESS-independent function for PERIOD proteins in the Drosophila clock. Neuron.

[pbio-0060183-b047] Brandes C, Plautz JD, Stanewsky R, Jamison CF, Straume M (1996). Novel features of drosophila period Transcription revealed by real-time luciferase reporting. Neuron.

[pbio-0060183-b048] Saez L, Meyer P, Young MW (2007). A PER/TIM/DBT interval timer for Drosophila's circadian clock. Cold Spring Harb Symp Quant Biol.

[pbio-0060183-b049] Myers MP, Wager-Smith K, Rothenfluh-Hilfiker A, Young MW (1996). Light-induced degradation of TIMELESS and entrainment of the Drosophila circadian clock. Science.

[pbio-0060183-b050] Taylor JW, Ott J, Eckstein F (1985). The rapid generation of oligonucleotide-directed mutations at high frequency using phosphorothioate-modified DNA. Nucleic Acids Res.

[pbio-0060183-b051] Crews ST, Thomas JB, Goodman CS (1988). The Drosophila single-minded gene encodes a nuclear protein with sequence similarity to the per gene product. Cell.

[pbio-0060183-b052] Saez L, Young MW (1996). Regulation of nuclear entry of the Drosophila clock proteins period and timeless. Neuron.

