# Supplementary material for: Activating PER Repressor through a DBT-Directed Phosphorylation Switch
Source: PLoS Biol. 2008 Jul 29;6(7):e183. doi: 10.1371/journal.pbio.0060183 (PMC2486307; doi:10.1371/journal.pbio.0060183)
Supplement: Figure S1 — Flies were entrained to an LD cycle for 3 d before testing. Thereafter, locomotor activity of individual flies was recorded in constant darkness at 25 °C. Representative actograms (top) and periodograms (bottom) for each genotype assayed are shown. For each periodogram, the lower limit of statistical significance (p < 0.01) is indicated by a slanted line. (112 KB PDF) [file pbio.0060183.sg001.pdf]

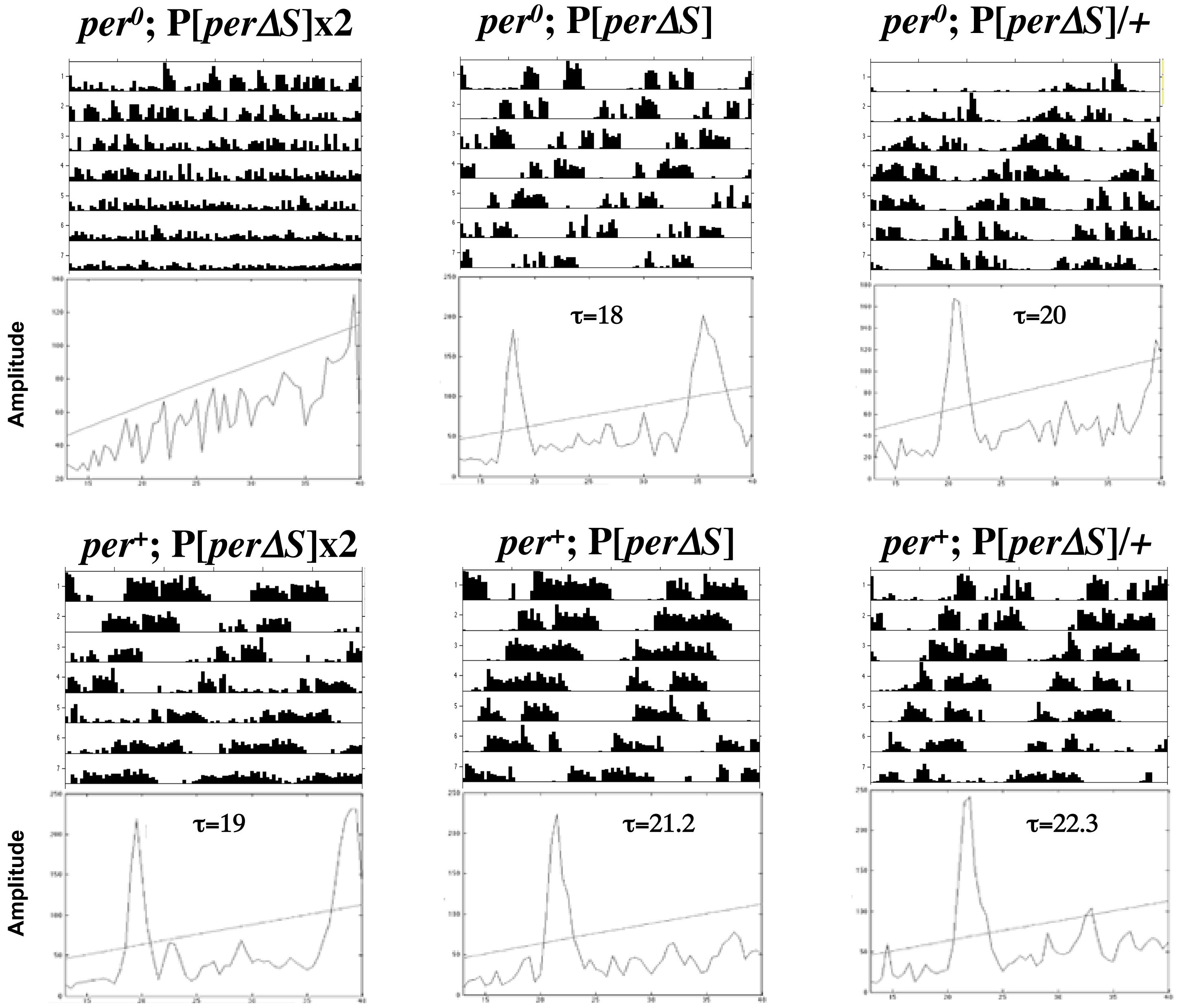
per0; P[perΔS]x2
per0; P[perΔS]
per0; P[perΔS]/+
τ=18
τ=20
Amplitude
per+; P[perΔS]x2
per+; P[perΔS]
per+; P[perΔS]/+
τ=19
τ=21.2
τ=22.3
Amplitude
