# Supplementary figures and images for: Activating PER Repressor through a DBT-Directed Phosphorylation Switch
Source: PLoS Biol. 2008 Jul 29;6(7):e183. doi: 10.1371/journal.pbio.0060183 (PMC2486307; doi:10.1371/journal.pbio.0060183)

**A**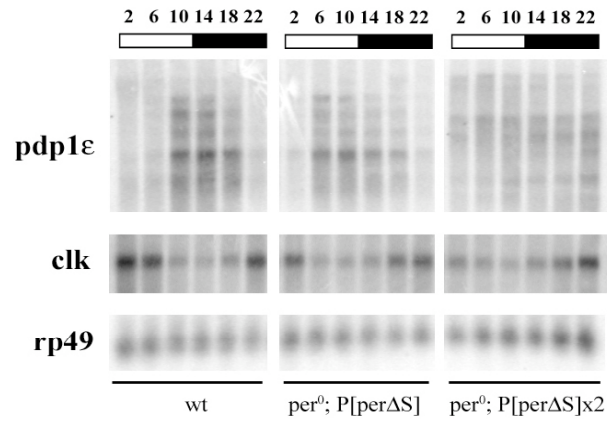**C**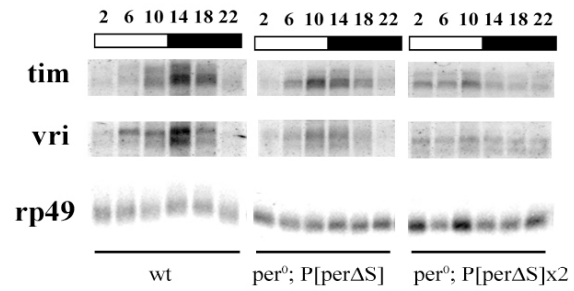**E**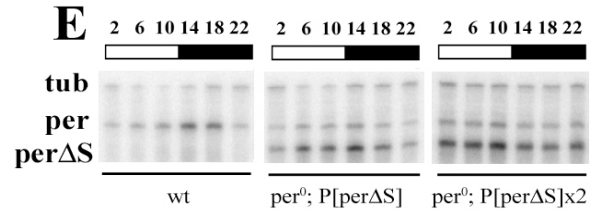**B**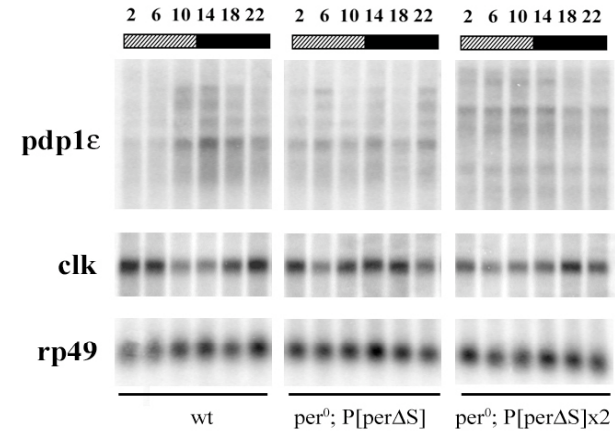**D**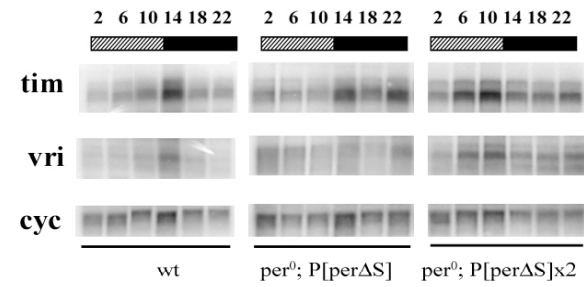**F**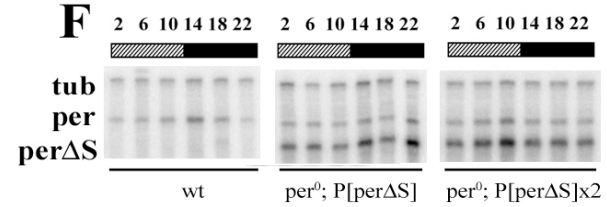

Supplement: Figure S2 — RNA samples were collected for 1 d in LD (indicated by altering open and closed horizontal bars in (A), (C), and (E)), and 1 d in DD (subjective day indicated by hatched bars, in (B), (D), and (F)). Genotypes are shown at the bottom of each panel, and RNA profiles assessed are indicated on the left. Constitutive tubulin, rp49, and cyc mRNAs were measured for normalization. (E) and (F) are the results of an RNAase protection assay. Top, middle, and bottom bands in (E) and (F) represent protected fragment for tubulin, endogenous per gene mRNA, and protected fragment from transgenic perΔS, respectively. Numbers indicate hours in a 24-h cycle when RNA was collected. (715 KB PDF) [file pbio.0060183.sg002.pdf]

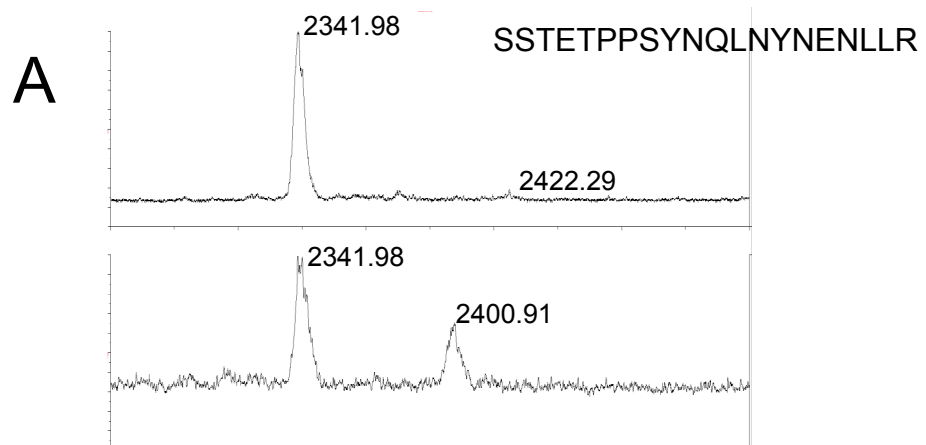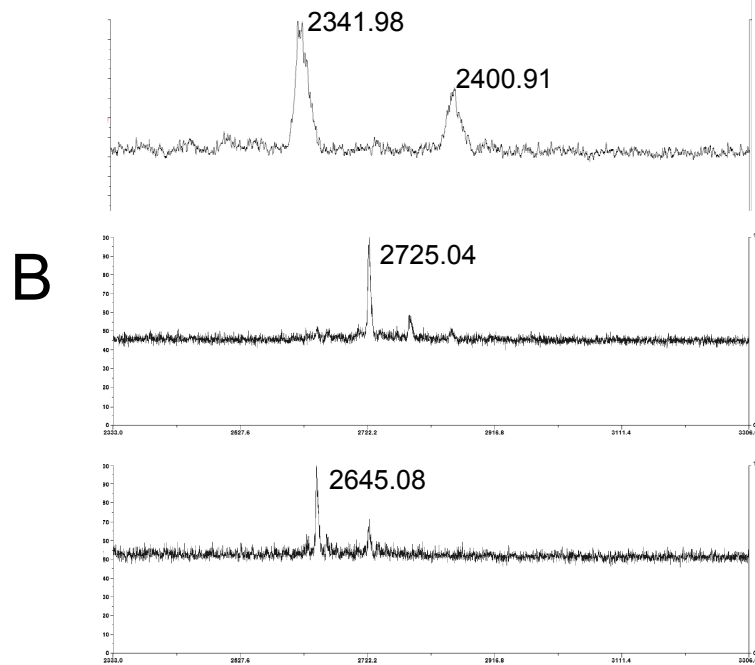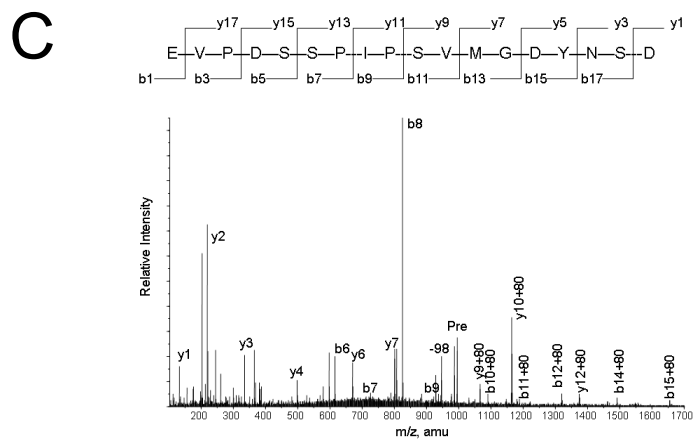

Supplement: Figure S3 — Recombinant PER fragments were individually phosphorylated with DBT and digested with trypsin. (A) and (B) were derived from peptide 606-SSTETPPSYNQLNYNENLLR-625. Each peptide fraction containing the majority of the radioactivity was treated with propanethiol (A) or phosphatase (B) and analyzed by mass spectrometry. The peptide masses of untreated (top) and treated samples (bottom) were compared to identify the phosphorylated residue(s). (A) shows an example of propanethiol treatment. Notice the change in the masses of the peptide 2422.29 after treatment due to a substitution reaction at the phosphorylation site, which reduces peptide mass by 21 Da per phosphorylated residue, improving ionization efficiency. (B) shows the results obtained following alkaline phosphatase treatment, leading to a reduction of peptide mass by 80 Da per phosphorylated residue. Peptide fractions that responded to both treatments were subjected to Edman sequencing to identify the position of individual phosphorylated amino acids in the peptides. (C) is a tandem mass spectrometry (MS/MS) spectrum of the PER peptide 1124-EVPDSSPIPSVMGDYNSD-1143, which is phosphorylated at position 10 (S-1134). The MS/MS spectrum shows a doubly charged ion at m/z 994.9 (Pre) corresponding to the mass of the phosphorylated peptide. A loss of 98 Da (H3PO4) was observed for this precursor and some fragment ions under low collision-induced dissociation. The peaks labeled y1–7 and b6–9 correspond to the masses of b and y fragment ions of unmodified peptides. The addition of 80 Da (mass of PO3) was observed for the b10 (b10+80) and y9 (y9+80) ions, demonstrating that phosphorylation was on the Ser at position 10. (335 KB PDF) [file pbio.0060183.sg003.pdf]
